# Foam-Based Wearable Devices Embedded with Shear-Thickening Fluids for Biomedical Protective Applications

**DOI:** 10.3390/ma19020391

**Published:** 2026-01-19

**Authors:** Oluwaseyi Oyetunji, Abolghassem Zabihollah

**Affiliations:** Mayfield College of Engineering, Tarleton State University, Stephenville, TX 76402, USA; oluwaseyi.oyetunji@go.tarleton.edu

**Keywords:** STF, foam, impact, adaptive response, protective brace

## Abstract

Falls are a leading cause of bone fractures among the elderly, particularly hip fractures resulting from side falls. This research deals with the feasibility of application of shear-thickening fluids (STFs) to design self-protective wearable devices to rapidly respond to sudden impact due to falls. The device consists of a lightweight, flexible foam structure embedded with STF-filled compartments, which remain soft during normal movements but stiffen upon sudden impact, effectively dissipating energy and reducing force trans-mission to the bones. First, a foam-based sandwich panel filled with STF is fabricated and subjected to several falling scenarios through a ball drop test. The induced strain of the device with and without STF is measured using Fiber Bragg Grating (FBG) sensors. Then, the effect of localized STF is explored by fabricating a soft 3D-printed (TPU) sandwich panel filled with STF at selected cavities. It was observed that the application of STF reduces the induced strain by approximately 50% for the TPU skin device and 30% for the foam-based device. This adaptive response mechanism offers a balance between comfort and protection, ensuring wearability for daily use while significantly lowering fracture risks. The proposed solution aims to enhance fall-related injury prevention for the elderly, improving their quality of life and reducing healthcare burdens associated with fall-related fractures.

## 1. Introduction

In today’s engineering world, materials that can intelligently adapt to their surroundings are becoming increasingly important, and their applications in various industries, from aviation to biomedicals and transportation, among many others, are now needed as never before due to their weight, flexibility, and compactness. In the medical industry, the exploration of smart material inclusiveness is increasingly gaining traction as it could help both patients and practitioners to quickly detect, report, and recommend necessary next lines of actions.

Falls are one of the most urgent problems of elderly people because they are the most common cause of injury and hospitalization in the aging community all over the world. Injuries related to falls concern over 37 million cases annually all over the world, are the leading cause of injury, morbidity, and mortality in older adults worldwide, and are a critical area of public health concern with wide social and economic impacts. It has been shown by epidemiology that approximately one out of three adults over 65 years old experiences a fall each year, and the risk increases with age and comorbidity [1,2,3,4,5]. In addition to age, health issues such as attention deficit hyperactivity disorder (ADHD) and sensory processing disorder (SPD) may cause falling due to losing balance in children [6]. Clinical evidence has demonstrated that fall-related injuries, including hip and ankle fractures, not only lead to a certain degree of physical disability, but also impair mobility and independence in the long term and cause deaths. This trend has generated an alarming need when it comes to protective solutions with a focus on biomechanical efficiency and user level of comfort in a day-to-day setting [7]. The financial cost of fall-related injuries is no less distressful. The expenditure to address falls and fall-related fractures in the elderly population will grow exponentially in line with demographic changes in terms of population aging. It is estimated that the United States alone (and aggregate estimates in Europe and Asia are on an increasing trend) spends over 50 billion dollars yearly on the medical expenses linked to falls of older adults [8,9]. These expenses include acute care medicinal expenses, medical surgery, rehabilitation, and long-term nursing care. Beyond monetary impacts are the psychological ones, which include fear of recurring falls, social seclusion, and quality of life, and the psychological implications continue to feed the problem. Scientists and researchers are providing solutions to fall-related injuries. The most common solution for this problem is prescribing protective braces. Protective devices such as rigid braces and foam paddings, as well as hip protectors, have conventionally been used to address the consequences of falls. They are, however, unwieldy, with limited ergonomic adaptability, and they tend to cause discomfort to users, affect mobility negatively, and interfere with the socialization process, thereby limiting compliance among older people [10]. To overcome the problem of fall-related injuries in adults, several protective systems were created, including orthoses to wear, hip protectors, and smart wear. Perhaps the most well-known of the prevention solutions in widespread use are hip protectors of either a rigid shell or a shock-absorbing material. They are created as a way of redistributing and dissipating the energy that comes with a fall, therefore minimizing the chances of the patient presenting with hip fracture. In a similar manner, braces and padding have also been used to help reduce local impact forces on specific areas of concern such as the hip, knee, and the ankle.

In recent years, wearable devices that incorporate sensors to measure falls and send notifications and provide an objection-monitoring aspect to aging care have also emerged [11]. The rather low adoption rate shows a fatal design constraint of not compromising wearability to provide good protection. Lightweight, flexible, and unobtrusive but impact-resistant protective systems are required to achieve consistency in their usage [12,13,14]. A look into fall-related injuries in the elderly population and sportsmen calls for innovative alternative approaches to solve fall-related injuries. Considering the multi-disciplinary nature, biomedical applications require materials capable of intelligent adaptation to environmental conditions.

**Figure 1:**
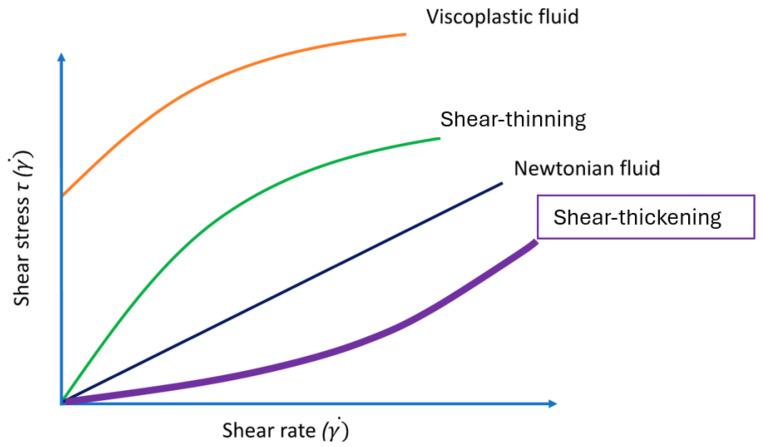
Shear stress vs. shear rate behavior for common fluids; Newtonian, viscoelastic, shear-thinning, and shear-thickening fluids.

Advances in materials science have opened a new horizon to develop new adaptive soft materials for biomedical applications. Soft materials are substances that exist in a state between solids and fluids, showing significant deformation and fluidity when subjected to external loads. Shear-thickening fluids (STFs), well-known soft materials, are non-Newtonian fluids. Figure 1 illustrates that the fundamental distinction between STFs and conventional non-Newtonian fluids lies in their dramatic performance transformation capabilities under applied stress.

The most common model describing the stress–strain behavior of STFs is the power law-based equation. The model explains the shear stress needed to shear a fluid at a given rate can be determined by the following equation.

(1) $$\tau =K\cdot {\dot{\gamma }}^{n}\;n>1$$

In the power law model, the value of *n* = 1 corresponds to a Newtonian fluid, while 0 < *n* < 1 is a shear-thinning (pseudoplastic) fluid and *n* > 1 represents a shear-thickening (dilatant) fluid.

The increase in shear rate causes a decrease in viscosity, and hence gradually leads to the minimum value of viscosity. Then, the fluid begins to experience an increase in viscosity slowly with further increase in shear rate, and there is a sudden increase in the viscosity, leading to change in phase from a liquid or fluid phase to a quasi-solid state [15].

**Figure 2:**
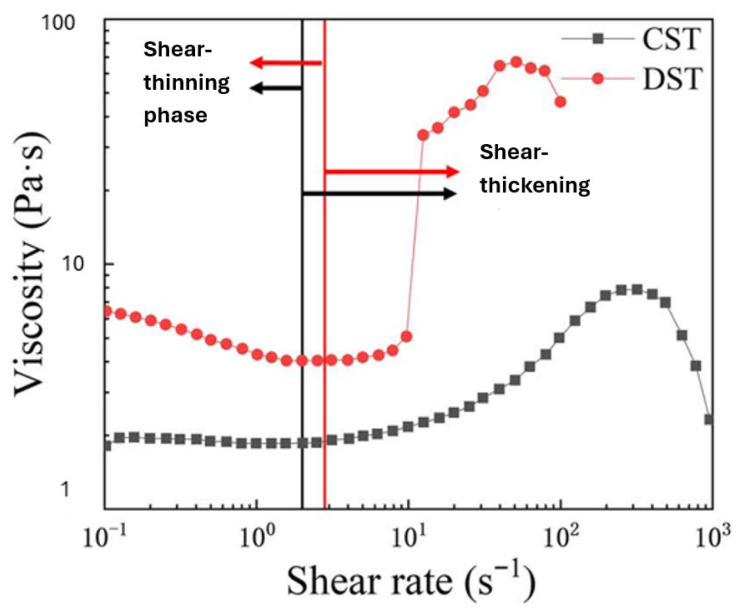
Continuous shear thickening and discontinuous shear thickening [16].

Figure 2 illustrates, on a logarithmic scale, the transition from shear thinning to shear thickening versus viscosity [16].

The complexity underlying STF originates from intricate particle-scale phenomena that cascade into macroscopic property changes. The stress–strain behavior of STFs is a combination of multiple concurrent mechanisms, ranging from hydrodynamic clustering to direct particle contact networks, that collectively govern system response characteristics [17,18,19].

**Figure 3:**
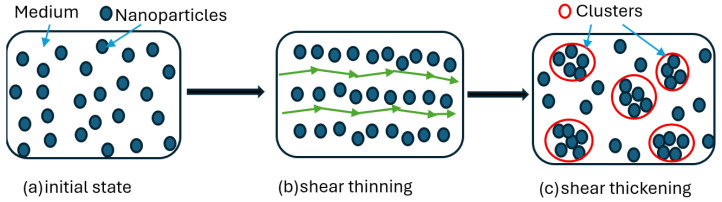
Shear behavior of colloidal suspensions; (**a**) at initial state, nanoparticles are randomly dispersed within the medium; (**b**) suspension is subjected to low shear rate and nanoparticles make a relatively regular arrangement allowing smooth flow; (**c**) at high shear rate, nanoparticles making clusters block smooth flow.

At low-speed strain rate, particles flow freely, while by increasing speed of loading, particles generate clusters that obstruct the flow, as shown in Figure 3.

Advances in nanoparticle synthesis methodologies, surface modification techniques, and computational modeling capabilities now enable systematic STF design rather than empirical-optimization approaches [20,21].

These sophisticated colloidal systems transcend traditional material limitations by embodying adaptive behavior principles, transitioning from free-flowing liquid states to quasi-solid configurations within milliseconds of encountering critical shear conditions [22].

This unique characteristic makes STFs excellent soft materials for applications where passive adaptability offers strategic advantages over static material solutions. The implications extend beyond simple viscosity modifications, enabling entirely new design philosophies where materials actively contribute to system performance rather than merely providing structural support.

Recent advances in additive manufacturing in three-dimensional printing with multiple material phases provide us with the opportunity to use 3D printing to fabricate materials composed of multiple materials phases, such as solids, fluids, and gels. As a result, STFs have become an interesting type of smart material for designing wearable devices with the capability to provide self-assistive support for sudden impact. Impact loads results in high-speed shear rate, which in turn leads to changing the stress–strain behavior of the STF as demonstrated in Figure 3 and thus results in dissipating dynamic energy during impacts [23,24]. This phenomenon has been utilized in many applications, such as sports protection and vibration damping where STF-impregnated fabrics and composites have shown increased energy absorption capability without jeopardizing flexibility [25,26]. Combined with textile or polymeric matrices, STFs promise the creation of hybrid metamaterials with the capability to provide excellent protection in low-profile protective formats. Research has shown an increase in energy absorption when silica-particle-based STF is incorporated into polyurethane [27,28]. A work by Galindo et al. [29] on cork composite structure with microchannels filled with STF showed an improvement in the panel’s performance under low-velocity impact.

This work intends to examine the feasibility of using STFs to develop a self-assistive wearable device aimed at minimizing fall-related injuries. First, an efficient production procedure is examined to produce STF in an efficient method. Then, a sandwich panel with different skin materials is designed and filled with pockets of STF. The performance of samples in absorbing energy is examined using a ball drop test. The changes in absorption energy are determined by measuring the change in strain of the skin using an array of Fiber Bragg Grating (FBG) sensors.

## 2. Materials and Methods

**Figure 4:**
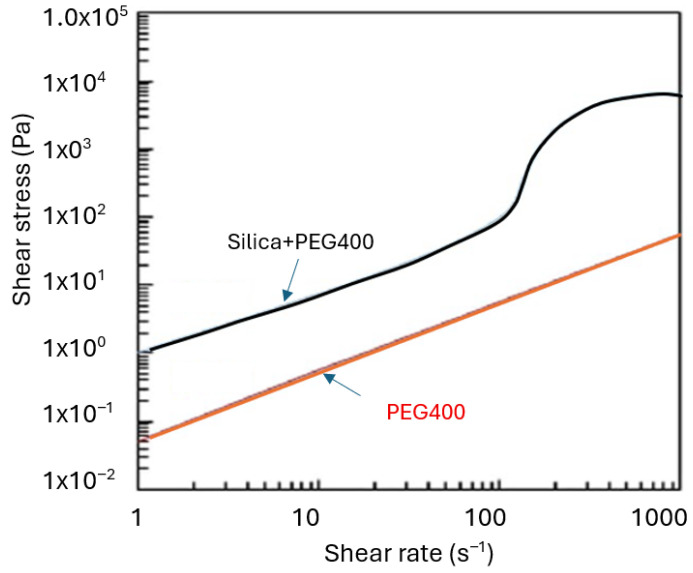
Shear stress vs. shear rate of STF and PEG (constructed based on [30]).

It is understood that STF can be produced by a variety of combinations. Polyethylene glycol (PEG) and silica are commonly used for engineering applications. Therefore, in this work, these two materials are used as the main ingredient to prepare STF. The nonlinear relationship between shear stress and strain rate for shear-thickening fluids is illustrated in Figure 4. As can be observed, adding silica significantly enhances the shear-thickening effect by increasing the shear rate.

Therefore, for testing performance and experimental work, PEG and silica have been used as the starting point to produce STF.

### 2.1. Sample Preparation

#### 2.1.1. Producing STF Material

The shear-thickening fluid composition was made to present the discontinuous shear-thickening properties. The STF was prepared using nanodisperse spherical fumed silica Silicon dioxide Nanopowder KH570 processing with an average diameter of 20 nm and a purity of 99%, procured from XFNANO, Nanjing, China. The fumed silica particles were dispersed in a carrier fluid of polyethylene glycol (PEG) manufactured by thermos scientific^®^, Waltham, MA, USA, with a molecular weight of 400 g/mol, and ethanol was used as solvent, manufactured by Uniclean^®^ America, Katy, TX, USA, with 99.7% purity. PEG is a non-flammable and involatile liquid, stable at room temperature [31].

To prepare the shear-thickening fluid, silica/PEG samples underwent mechanical mixing and ultrasonication techniques at weight fractions of 25%, as provided in Table 1. It is noted that the weight fraction of 25% was selected based on parametric studies conducted by Astaraki et al. [32,33].

**Figure 5:**
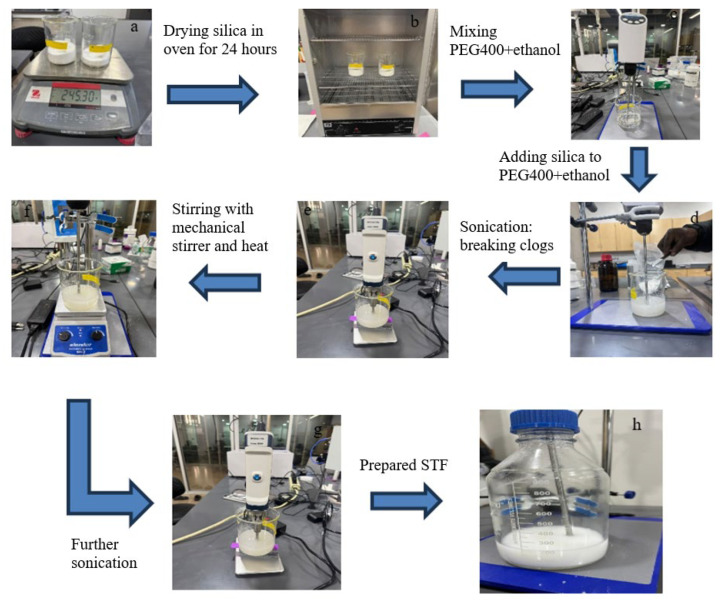
STF preparation procedures: (**a**) weighing of silica fume, (**b**) fumed silica drying for 24 h @ 60 °C, (**c**) mixing PEG400 with ethanol, (**d**) adding silica to the solution of PEG400 and ethanol, (**e**) sonication, (**f**) stirring again after sonication, (**g**) ultrasonication, (**h**) prepared shear-thickening fluid.

It is important to note that silica fumes are sensitive to moisture. Several tests were carried out to achieve optimal state of the nanoparticles; first, the nanoparticle was weighed (Figure 5a) and then placed in an evaporator 24 h at 60 °C (Figure 5b). The preparation moves to the mixing of ethanol and PEG400 after the dried silica fume was removed from the evaporator in a ratio of 3:1 using a mechanical stirrer (LAB FISH, Zhejiang Huili Experimental Instrument Co., Ltd., Huzhou, China purchased through LAB FISH-US) (Figure 5c) at 500 rpm running at normal room temperature for 60 min to form a homogenous solution. For free particle distribution and aggregation, the dried silica fume was gradually added to the solution (Figure 5c) to avoid excessive clogs in the shear-thickening fluid formation (Figure 5d). The mixture was placed under an ultrasound sonification device manufactured by Bonvoisin (model 8M25041104, power: 300 W, Hangzhou Yatuo Electronic Technology Co., Ltd., Hangzhou, China) for 3 h at 19 volts for 5 min intervals (Figure 5e). The ultrasonic device was used to break the bubbles in the mixture. Afterward, the mixture of silica fume, PEG400, and ethanol was stirred for 18 h at room temperature with a mechanical stirrer and then underwent magnetic stirring with heat for another 8 h at a low heat. Finally, the prepared STF was placed in an evaporator at room temperature for 10 h to remove remaining bubbles from the solution.

#### 2.1.2. Fabricating Sandwich Skins

**Figure 6:**
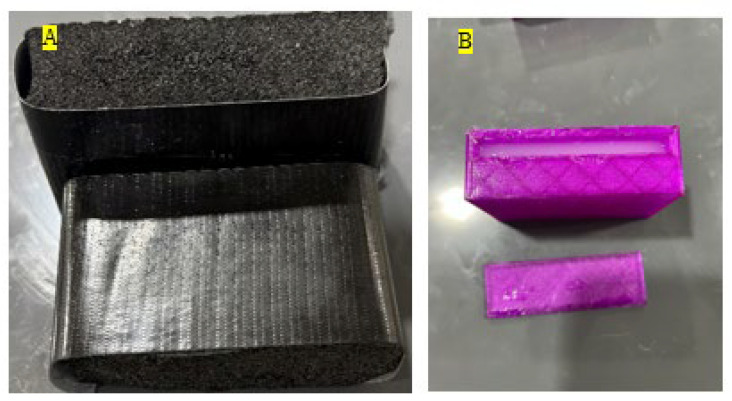
Test specimen; (**A**) foam core and (**B**) 3D-printed TPU.

Two types of base material, namely foam-based and a 3D-printed box made of the soft material Thermoplastic Polyurethane (TPU) with cavities to hold STF, are designed as shown in Figure 6 for conducting the ball drop testing. It is noted that these materials are commonly used as base materials for impact protection in many applications.

### 2.2. Experimental Setup and Testing

**Figure 7:**
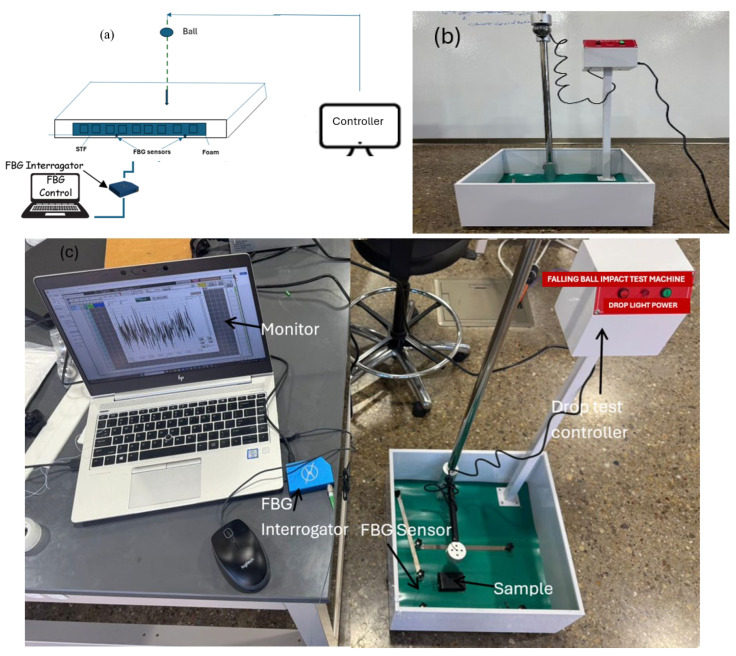
(**a**) Schematic diagram of experimental test, (**b**) steel impact drop tester, and (**c**) experimental setup for impact testing.

To study the feasibility of the application of STFs in energy absorption when falling, an in-house experimental setup was developed. Figure 7a shows the schematic diagram of the experimental test. The setup consists of a ball drop test machine to simulate the impact due to fall, an array of FBG sensors (FBG-MR0010, purchased from Micronor Sensors, Inc., Ventura, CA, USA) attached to the bottom of the samples to measure the strain, and an FBG interrogator to visualize and record the data in real time. For more details on principles of strain measurement using FBG, one may consult the article written by Zabihollah and Shi [34].

The ball drop machine is manufactured by GOYOJO with dimensions of 42 × 30 ×12 inches with varying adjustable drop height, an accuracy of 1 mm, and ball weights as shown in Figure 7b. Two types of base materials, foam and TPU, were used for making samples. First, the unfilled foam samples were used to study impact dissipation in the natural state. Then, the samples were filled with STF and subjected to the same impact loading to study the effect of adding STF on dissipating impact load. The impact tester balls were held by magnets using the controller, with the test sample for different drops placed in the overhead ball drop. Connected to the sample is the fiber optic cable and FBG interrogator for data acquisition. Figure 7c shows the complete experimental setup for ball drop testing.

## 3. Results

The impact test gives details about the STF impact absorption within the material. The impact drop test was an experiment to determine the efficacy of STF impact dissipation in load settings. A comparison of each response was investigated for impact testing and different behaviour. It is noted that the energy applied to a human ankle depends on the weight and height of the body. For example, for a 70 kg body, the energy applied to the ankle is approximately 70 J. It is understood that this amount increases for a basketball player when falling after jumping. Due to laboratory limitations, the results provided in this experimental work are scaled down 50 times.

The impact energy associated with the different drop heights was first calculated for all test masses (55 g, 64 g, 110 g, and 225 g) and their corresponding impact velocities were also calculated. Table 2 provides the corresponding impact velocities and impact energies for dropping heights between 100 mm and 500 mm for 55 g, 225 g, and 500 g. Because the drop height determines the free-fall speed, all masses follow the same trend: impact velocity increases from 1.40 m/s at 100 mm to 3.13 m/s at 500 mm, while the energy ranges from 0.05 J for 55 g balls drops from 100 mm to 2.2 J for 500 g balls drops from 500 mm.

### 3.1. Effect of Base Material on Impact Response

**Figure 8:**
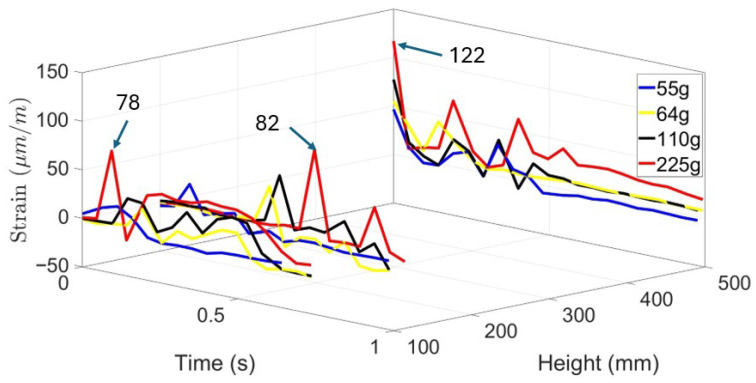
Low-velocity impact response of foam-based material using ball drop.

To investigate the effect of base material on impact response, each sample, foam-based and TPU, were subjected to various impact loads. Knowing that impact load is proportional to the potential energy of falling balls, different ball weights, namely, 55 g, 64 g, 110 g, and 225 g were dropped from different heights, 100 mm, 200 mm, and 500 mm. The heights were selected to simulate selected safe scenarios of human falls. Figure 8 provides the results of impact loads on strain response of foam-based samples without STF for drop heights of 100 mm, 200 mm, and 500 mm, respectively. It is observed that by increasing the drop height and the ball weight, strain at the bottom of the sample increases. One may also note that the magnitude of strain represents the stress applied to the body covered by the wearable device.

Upon a close look at the graphs, one may observe that strain signals follow several cycles before completing damping. This phenomenon is due to rebounding balls when hitting the samples and losing the samples.

**Figure 9:**
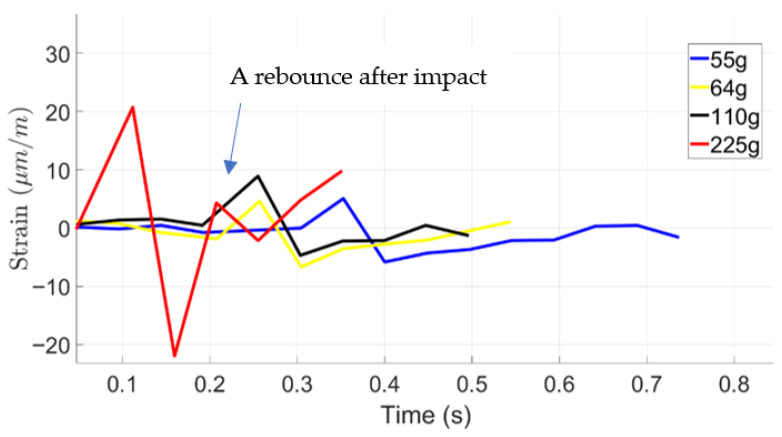
Graph of low-velocity impact tests with a 100 mm drop height for TPU.

**Figure 10:**
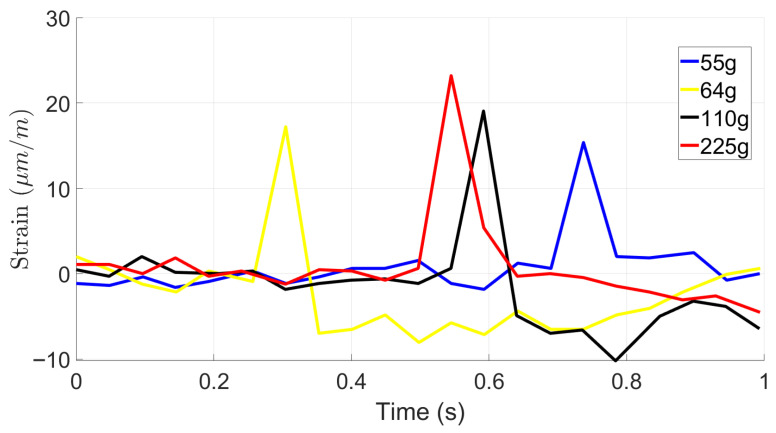
Graph of low-velocity impact tests with a 200 mm drop height for TPU.

**Figure 11:**
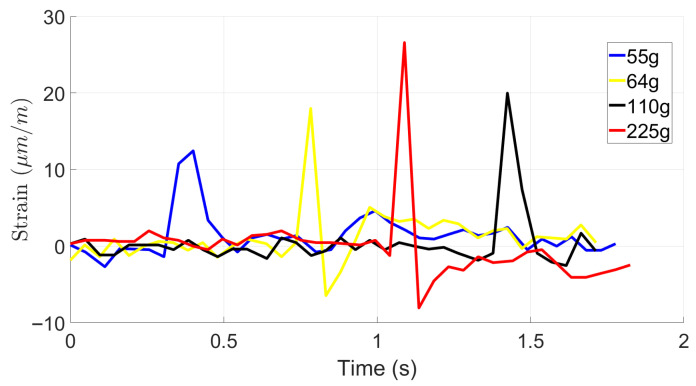
Graph of low-velocity impact tests with a 500 mm drop height for TPU.

Figure 9, Figure 10 and Figure 11 provide the results of impact loads on strain response of foam-based samples without STF for drop heights of 100 mm, 200 mm, and 500 mm, respectively. Similarly to the results of foam-based samples, it is observed that by increasing the drop height and the ball weight, strain at the bottom of the sample increases. However, the maximum value of induced strain for TPU base samples is approximately 70% lower than that of foam-based samples. This observation is expected as the TPU is stiffer than foam and requires more energy to deform.

Once again, one may observe that strain signals follow several cycles before completing damping. This phenomenon is due to rebounding balls when hitting the samples and losing the samples.

Table 3 provides the peak strain induced on the specimens made with foam and TPU skin. It is observed that in certain cases, strain induced on TPU is approximately one-third of that of the foam-based element. In addition, increasing the drop height and drop weight increases the induced strains.

### 3.2. Effect of STF on Impact Energy Absorption

In the next step, all samples were filled with STF material and subjected to similar ball drop tests. Knowing that the strain energy is proportional to changing the induced strain after impact (*u* = ∫*σdε*), the effect of STF on energy absorption can be determined by measuring the induced strain. The strain at the bottom of the element under impact is directly measured using surface-mounted FBG sensors. On the other hand, the input energy of the drop test is determined in Table 2.

**Figure 12:**
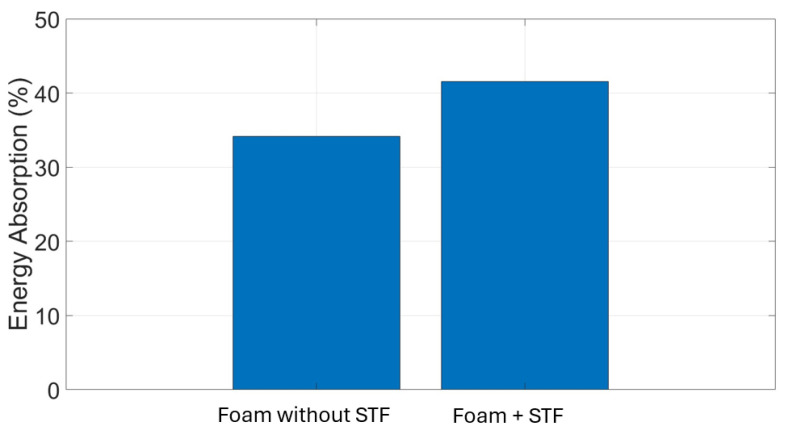
Energy absorption for 55 g of foam with/without STF for 100 mm.

Figure 12 shows that incorporating STF into the foam led to a modest reduction in the transmitted impact.

The STF-embedded foam exhibited a noticeable increase in energy absorption compared with the untreated foam. Under identical impact conditions, the foam without STF absorbed approximately 34% of the input energy, whereas the STF-treated foam demonstrated a higher energy absorption of about 41%, corresponding to an improvement of roughly 15–20%. The introduction of the shear-thickening fluid within the skin material contributed to the additional energy absorption.

**Figure 13:**
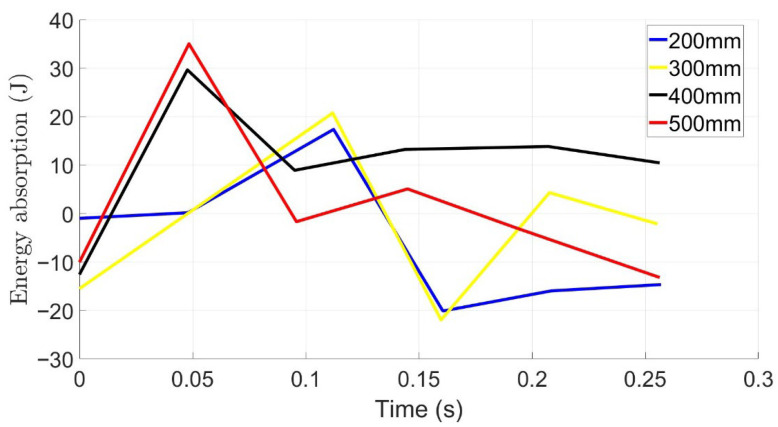
Energy absorption for TPU skin element filled with STF.

Figure 13 shows the energy absorption for the skin material TPU filled with STF under different drop heights ranging from 200 mm to 500 mm for a mass of 225 g. A damping reduction was observed at all drop heights under the impact load. At 200 mm, an energy absorption of 18 J was observed and twice that for the 500 mm using the same impact load. Comparing this with the figure above for the foam and TPU, the damping for the skin material filled with STF is faster and quicker. This combination is inherently stiffer and returns to equilibrium quickly following impact. As a result, it transmits minimal residual strain to the FBG sensor, indicating effective attenuation of post-impact motion.

## 4. Discussion

This experiment investigates the impact response of foam-based and TPU materials when subjected to drop tests from various heights using objects of different masses. The objective was to evaluate the material’s deformation characteristics and impact resistance, providing a baseline for comparison with shear-thickening fluid (STF). The test involved dropping weights of 55 g, 64 g, 110 g, and 225 g from heights of 100 mm, 200 mm, and 500 mm. Strain variations over time were recorded to assess the material’s deformation behavior and recovery after impact. Results showed that foam without STF exhibited greater strain amplitudes than foam impregnated with STF at all test conditions, reflecting its higher deformability and lower stiffness. At a drop height of 100 mm, the maximum strain reached approximately 120 µm/m under a 225 g impact, whereas lighter weights produced lower strain peaks between 25 µm/m and 40 µm/m. As drop height increased, strain magnitude also increased in a nonlinear manner. At 200 mm, multiple strain peaks appeared, indicating energy dissipation through rebound and internal wave propagation. The foam’s open-cell structure facilitated compression under impact, followed by partial elastic recovery. The highest strain values were observed at 500 mm, where the 225 g mass produced strain amplitudes exceeding 110 µm/m. The oscillatory pattern following each impact highlights the foam’s viscoelastic response, a typical behavior for porous materials capable of both elastic and viscous deformation. When compared directly, foam without STF showed higher deformation and slower relaxation, while the foam with STF exhibited lower deformation and faster recovery. Foam absorbs impact energy primarily through large-scale compression, resulting in greater strain amplitudes and prolonged recovery times. These findings underscore the distinct impact absorption mechanisms of both materials. The contrasting mechanical behaviors suggest that combining foam with STF could yield protective structures that achieve an optimal balance between comfort, flexibility, light weight, and impact resistance. In general, adding STF reduced the impact effect by ~25%.

The results for the TPU base material show similar trends of energy absorption. However, for the TPU base material, the induced energy at similar impact load is approximately 70% lower than that of the foam-based material.

## 5. Conclusions

The feasibility of using shear-thickening fluids for biomedical applications, specifically in wearable self-protective braces were studied. An efficient yet functional recipe was introduced for preparation of STF. Two carrier bodies, namely foam and TPU, were prepared to prepare the test specimens for investigating the impact-resistance behavior of wearable devices filled with STF. This research has the following conclusions:

- STFs enhance the dynamic stiffness of flexible substrates such as foams and fabrics, leading to reduced deformation amplitudes during impact.
- The substantial reduction in strain-energy index for foam + STF in this work mirrors the improvements reported in impact-protective foams, where STF enhances load distribution and limits bottoming-out effects.
- The rheological behavioral tests for the shear-thickening fluids showed a significant increase with viscosity as the shear rate increased.
- The TPU impregnated with STF exhibits a great performance due to greater deformation and energy dissipation.
- The brace with TPU skin shows higher capacities for absorbing higher-impact velocities.
- The behavior of TPU + STF positions the material as a promising candidate for biomedical wearable devices, especially in applications aimed at fall mitigation and impact protection.
- The application of STF reduces the induced strain by approximately 50% for TPU skin device and 30% for foam-based device.
- The present wearable self-protective device offers a balance between comfort and protection, ensuring wearability for daily use while significantly lowering fracture risks.

This research needs to be extended to explore the effect of the lumped size and location of STF at selected locations to develop customized self-protective wearable devices for desired applications and settings.

## Figures and Tables

**Table 1:** Material properties used to prepare STF.

Material	Chemical Formula	Density (g.cm^−3^)	Molar Mass (g.mol^−1^)	Weight Fraction
Silica	SiO_2_	2.2	60.09	25 g
PEG400	C_2n_H_4n+2_O_n−1_	1.128	400	66.5 mL
Ethanol	C_2_H_6_	0.789	45.068	

**Table 2:** Impact velocities and energies for drop heights.

Drop Height (mm)	Velocity (m/s)	Energy (J)		
		55 g	225 g	500 g
100	1.40	0.05396	0.22073	0.490
200	1.98	0.10791	0.44145	0.883
300	2.43	0.16187	0.66218	1.324
400	2.80	0.21582	0.88290	1.765
500	3.13	0.26978	1.10363	2.207

**Table 3:** Strain induced on specimen vs. height and weight, (µm/m).

Height, mm	Weight, g					
	55		110		225	
	Foam	TPU	Foam	TPU	Foam	TPU
100	20	6	28	8	78	21
200	26	12	52	19	82	24
500	48	14	76	20	122	27

## Data Availability

The original contributions presented in this study are included in the article. Further inquiries can be directed to the corresponding author.
